# Evaluation of blood culture media for the detection of fungi

**DOI:** 10.1007/s10096-014-2218-4

**Published:** 2014-08-08

**Authors:** U. Nawrot, B. Kowalska-Krochmal, B. Sulik-Tyszka, M. Kozak, K. Świętek, M. Pajączkowska, E. Piątkowska, D. Rosiak, E. Swoboda-Kopeć

**Affiliations:** 1 Department of Microbiology, Medical University of Wrocław, Wrocław, Poland; 2 Department of Medical Microbiology, Medical University of Warsaw, Warsaw, Poland; 3 Department of Diagnostics, Laboratory of Microbiology, Military Hospital, Wrocław, Poland; 4 Department of Dental Microbiology, Medical University of Warsaw, Warsaw, Poland

## Abstract

The aim of this study was to compare the utility of BACTEC™ Mycosis-IC/F (Mycosis), BACTEC™ Plus Aerobic/F (Aerobic), and BACTEC™ Plus Anaerobic/F (Anaerobic) media in the detection of fungi from simulated (obtained by the inoculation of tested media first with sterile sheep’s blood and subsequently with one of 60 clinical yeast isolates) and clinical blood samples, taken during routine diagnostic examination in two hospitals. All tested strains grew on Mycosis as well as Aerobic bottles, and the time to detection obtained for Mycosis was significantly shorter (*p* < 0.05). The largest differences in the time to positivity was found for *Candida glabrata* and *Cryptococcus neoformans*, when Mycosis preceded Aerobic in 20–48 h (mean 35.5 h) and 0.7–64 h (mean 24 h), respectively. On the contrary, *C. krusei* were detected earlier in Aerobic media. In clinical samples, the detection of *C. glabrata* was also significantly faster in Mycosis than in Aerobic (29.22 ± 11.48 h compared to 86 ± 40 h). The media complement each other and, in 45 % of clinical examination sets, a single positive medium was noted (25 % in Mycosis and 19 % in Aerobic). The study proved that both Aerobic and Mycosis media serve as the correct condition for the culture of fungi and that they varied significantly in the detection time of clinically important species. This result could suggest that the simultaneous use of Aerobic as well as Mycosis media may improve the time of diagnosis in many patients, especially those infected with *C. glabrata* or *C. neoformans*.

## Introduction

A growing number of fungemia cases have been observed in recent decades and *Candida* species have been placed as the fourth most common microorganism isolated from blood samples [1–3]. It is supposed that many cases of invasive mycoses, including invasive candidosis, remain undiagnosed, mostly because of the insufficient sensitivity of blood cultures, which has been estimated as 50 % [3, 4]. In practices, many patients have been successfully treated empirically. However, because of the significant epidemiological changes observed in recent decades, and the increase in the rate of infection due to *C. glabrata*, *C. krusei*, or *C. parapsilosis*, which are frequently resistant to azoles or echinocandins, such empirical therapy may not necessarily hit causative pathogens. In order to improve the early diagnosis of invasive fungal diseases, several serological tests detecting fungal biomarkers (antigens, DNA, or specific antibodies) have been developed, of which only a few have gained acceptance for routine diagnostics (glucan, mannan, anti-mannan) [3]. The results of each serological test must be interpreted with caution due to the risk of false results. The culture of fungi from normally sterile tissue, including blood, remains an essential diagnostic test for invasive mycoses. The isolation of causative pathogens, followed by their subsequent identification and susceptibility testing, enables implementation of a proper therapy and has a direct impact on the patient’s recovery.

According to the recommendations of the European Society for Clinical Microbiology and Infectious Diseases Fungal Infection Study Group (EFISG), blood from patients with suspected candidemia should be cultured in aerobic and anaerobic media, to allow the growth of typical bacterial as well as fungal pathogens. The EFISG emphasized that the culture sensitivity and time to positivity may vary, depending on the automatic blood culture systems and causative species [3]. One of the most widely used validated blood culture systems is BACTEC 9240 (Becton Dickinson, USA). It was postulated that this system can be less sensitive in the detection of some fungi, depending on the medium used [3]. The addition of BACTEC™ Mycosis-IC/F (Mycosis), a selective medium for fungi, to the standard set of aerobic and anaerobic bottles, e.g., BACTEC™ Plus Aerobic/F (Aerobic) and BACTEC™ Plus Anaerobic/F (Anaerobic), is thought to be the best method to enhance the sensitivity of fungal detection. However, due to the low frequency of fungemia and costs enhancement, many medical centers limit the practical use of such a specialized medium. The presented study aimed to evaluate the utility of the application of the Mycosis medium together with Aerobic and Anaerobic BACTEC media, based on the analysis of simulated as well as clinical blood samples.

## Materials and methods

### Strains

The study was performed on four *Candida* strains from the American Type Culture Collection (*C. albicans* ATCC 90028, *C. parapsilosis* ATCC 90018, *C. krusei* ATCC 6258, *C. glabrata* ATCC 90030) and 60 clinical blood isolates from the laboratory collection of the Department of Microbiology, Medical University of Wrocław, Wrocław, Poland (Table 1). The strains were preserved in 40 % glycerol at −80 °C. The 24–48-h-old cultures on Sabouraud agar medium were used to prepare a suspension of density 0.5 MF (in PBS), which was subsequently diluted (10^−2^, 10^−2^) to obtain final concentrations of 2–3 × 10^2^ CFU/mL. The final number of cells was tested by quantitative cultures on Sabouraud agar plates.

**Table 1:** Time to detection (TTD) of simulated fungal blood cultures obtained in Mycosis and Aerobic media

Species (number of strains)	Mycosis		Aerobic		Difference^a^	Wilcoxon signed-rank test
	Mean (h) (±SD)	Range	Mean (h) (±SD)	Range	Mean (h) (±SD)	
*C. albicans* (14)	26.6 (5.6)	20–39	27.36 (4.6)	20.5–39.33	−0.75 (3.07)	*p* = 0.18
*C. parapsilosis* (12)	31.41 (3.41)	26.7–41	30.5 (4.21)	27.7–44.3	0.91 (2.39)	*p* = 0.06
*C. glabrata* (8)	21.36 (2.4)	19–25.5	56.9 (7.95)	44.2–70.3	−35.5 (7.46)	***p*** * = 0.0006*
*C. krusei* (6)	21.07 (1.17)	18.5–23.7	19.09 (0.9)	17.5–21	1.97 (1.63)	***p*** ** = 0.004**
*Candida* spp.^b^ (7)	41.03 (20.77)	19.2–75.8	36.57 (16.64)	18.2–69.2	4.46 (8.55)	***p*** ** = 0.03**
*Cryptococcus neoformans* (8)	55.24 (10.9)	42.5–77.3	78.27 (23.4)	47.8–113.3	−24 (24.1)	***p*** ** = 0.0005**
Others^c^ (5)	33.43 (11.6)	24.6–59.7	37.06 (18.6)	25.17–76		*p* = 0.5
Total (60)	32.4 (13.9)	18–77.3	39.48 (21.8)	17.5–113.33	−7 (16.8)	***p*** ** = 0.017**

^a^Difference between TTD obtained in Mycosis and Aerobic media (Mycosis minus Aerobic)

^b^ *Candida* spp. include *C. guilliermondii* (3), *C. kefyr* (2), *C. spherica* (1), and *C. lipolytica* (1)

^c^Others include *Blastoschizomyces capitatus* (3), *Trichosporon asahii* (1), and *Exophiala* spp. (1)

### Preparation of simulated blood culture and processing

The blood culture bottles BACTEC™ Mycosis-IC/F (Mycosis), BACTEC™ Plus Aerobic/F (Aerobic), and BACTEC™ Plus Anaerobic/F (Anaerobic) were first inoculated aseptically with sterile sheep’s blood (5 mL per bottle) and subsequently with 50 μL of fungal suspension containing 10–20 fungal cells (mean final density: 0.41 CFU/mL). The samples were immediately placed in the compartment of the BACTEC 9240 and incubated until positivity (5 days for Aerobic and Anaerobic, and 14 days for Mycosis). During each experiment, the strains were tested in triplicate. After incubation, positive as well as negative bottles were subcultured on Sabouraud agar, CHROMAgar Candida, and Columbia agar.

Statistical analysis of the results was performed with the use of STATISTICA software. The positivity rate of a particular media was compared by Fisher’s exact test. For the comparison of the time to detection (TTD), the Wilcoxon matched-pairs signed-rank test and the *t*-test for differences between means for independent variables were used. Values of *p* < 0.05 were considered as being statistically significant.

### Analysis of the results of clinical blood samples testing

The results of blood cultures performed routinely in two clinical hospitals were analyzed retrospectively. The first hospital (Center I) analyzed a period of 26 months (January 2012 to February 2014), while the second (Center II) analyzed a 12-month period (2013). The patients with a clinical indication for blood culture testing [e.g., fever with systemic inflammatory response syndrome (SIRS], organ and systemic infections, postoperative infections, urinary tract infections, pneumonia, meningitis, endocarditis, catheter-related infections, etc.] were examined according to the standard protocols. The analysis was limited to the examination sets cultured from Aerobic, Anaerobic, and Mycosis bottles, taken at the same time (one after another) and from the same place (venipuncture or catheter). In both hospitals, inoculation of the media with 8–10-mL blood aliquots was recommended; however, the data regarding the real blood volume in particular samples was not collected. The blood cultures were transported to the laboratory in a thermo bag within 1–10 h and subsequently incubated in the BACTEC 9240 compartment. Center I incubated Aerobic media for up to 7 days, Anaerobic for up to 10 days, and Mycosis for up to 14 days. Center II applied 5 days (for Aerobic and Anaerobic) or 7 days (Mycosis) incubation protocols. Cultured microorganisms were identified with the use of the VITEK®2 Compact (bioMérieux) system and tests.

## Results

### Bottles spiked with fungi

All the tested ATCC strains, as well as clinical isolates, grew on Mycosis as well as Aerobic bottles and no false-positive or false-negative result was found in these media. The ATCC strains were tested in three experiments (nine vials per strain). The TTD of *C. albicans* ATCC 90028 (28.66 ± 1.82 h in Mycosis and 29.7 ± 2.04 h in Aerobic), *C. glabrata* ATCC 90030 (21.66 ± 0.817 h in Mycosis and 57.37 ± 2.22 h in Aerobic), and *C. parapsilosis* ATCC 90018 (31.16 ± 0.577 h in Mycosis and 30.58 ± 0.82 h in Aerobic) were placed among the range of TTD results obtained for clinical isolates of these species (Table 1). In contrast, the detection of *C. krusei* ATCC 6258 was significantly delayed compared to clinical blood isolates of *C. krusei*, which was noticeable in Mycosis (24.46 ± 1.44 h vs. 21.07 ± 1.17 h for clinical isolates; *p* < 0.001; *t*-test for independent variables) as well as in Aerobic media (28.6 ± 2.8 h vs. 19.09 ± 0.9 h for clinical isolates; *p* < 0.0001; *t*-test for independent variables). A total number of 210 Mycosis and 210 Aerobic bottles were used to test clinical isolates (3–6 vials per single strain). The variability among the TTD results obtained for a single strain in the same medium was low (differences in the range 0–2.5 h). The most quickly detected species were *C. kefyr* (2 isolates; 19.79 ± 0.629 h in Mycosis and 19 ± 1.1 h in Aerobic) and *C. krusei*, which were detected about 2 h earlier in Aerobic (19.09 ± 0.9 h) than in Mycosis media (21.07 ± 1.17 h) (*p* < 0.01; Wilcoxon test). Incubation of more than 1 day was required to detect isolates of *C. albicans*, *C. parapsilosis* (Table 1), and *C. guilliermondii* (3 isolates; 35.35 ± 2.07 h in Mycosis and 33.35 ± 1.12 h in Aerobic), as well as *Blastoschizomyces capitatus* (3 isolates; 29.2 ± 3.6 h in Mycosis and 28.47 ± 4.56 h in Aerobic) and *T. asahii* (1 isolate; 28.2 ± 2.6 h in Mycosis and 27.8 ± 3.2 h in Aerobic). In the case of *C. glabrata*, the detection was quick in the Mycosis medium, in the range 19–25.5 h and about 35.5 h longer in the Aerobic medium (range 44.2–70.3 h) (*p* < 0.001). Statistically significant differences in the TTD were also observed for *Cryptococcus neoformans* (Mycosis overtook Aerobic in about 1–2 days), which represents the slowest growing species (Table 1). With the exception of *C. neoformans*, the highest TTD values were observed in the cultures of rare fungal species, including *C. lipolytica* (67.5 ± 1.4 h in Mycosis and 67.42 ± 2.47 h in Aerobic), *C. spherica* (74 ± 2.5 h in Mycosis and 50.4 ± 2.9 h in Aerobic), and *Exophiala* sp. 58.9 ± 1.06 h in Mycosis and 75.25 ± 1.06 h in Aerobic. The analysis of the detection time for the whole population of blood isolates indicated statistically better results for Mycosis media (mean difference 7 h, *p* < 0.05).

In summary, the cultures on Mycosis media were positive within 24 h for 23 (38.3 %) and within 48 h for 49 (81.6 %) isolates. Positive Aerobic cultures were detected within 24 h for 15 (25 %) and within 48 h for 41 (68 %) isolates. Forty-two percent (25/60) of isolates were detected within 24 h on at least one of these media.

In the case of the Anaerobic medium, the detection of microbial growth by the BACTEC system was observed in 16 out of 180 vials (9 %), including 9/24 (37.5 %) bottles inoculated with *C. glabrata*, 5/42 with *C. albicans*, 1/6 with *C. guilliermondii*, and 1/3 with *T. asahii*. The TTD observed for *C. glabrata* ranged between 21.5 and 28.8 h (mean 26.02 ± 3.34) and was lower than those obtained in Aerobic media (*p* = 0.000; *t*-test). After the subculture of negative vials on solid media, fungal growth was found in 19/164 (11.58 %) samples spiked with the following species: *C. glabrata* (12/15), *C. krusei* (2/18), *C. albicans* (4/37), and *Cryptococcus neoformans* (1/24).

### Clinical blood samples positive for fungi

The analysis involved 51 sets of Aerobic, Mycosis, and Anaerobic blood cultures taken at the same time and resulted in at least one medium that was positive for fungi. With the exception of five examinations (from two patients) performed during antifungal therapy with echinocandins, blood culture sets were performed at the beginning of infection and before introducing targeted antifungal therapy. Blood samples originated from 28 patients, seven of which tested positive for concomitant bacterial infection. The causative fungal species were identified as *C. glabrata* (20 examination sets from 11 patients), *C. parapsilosis* (15/7), *Saccharomyces cerevisiae* (11/7), *C. albicans* (3/2), and *C. tropicalis* (2/1). Thirty-five out of 51 analyzed examination sets originated from 20 patients hospitalized in four departments (three surgical and one intensive care) from Center I. Among yeast species isolated in this hospital, *C. glabrata* (9 patients) and *S. cerevisiae* (7 patients) were dominant. Center II reported on 16 examination sets from eight patients of a single intensive care unit (ICU). Aerobic medium was positive in 13 and Mycosis in 11 examination sets, and the species isolated were *C. parapsilosis* (6 patients) and *C. glabrata* (2). Altogether, the highest yeast-positivity ratios were shown by Mycosis (80.3 %) and Aerobic (72.5 %) media, while the positivity of Anaerobic was significantly lower (25.49 %; *p* = 0.0000) (Table 2). In 13 examination sets (25.4 %), fungi were grown exclusively on Mycosis media, and in the next ten (19 %), the only positive medium was Aerobic. No statistically significant differences were observed in the positivity ratio related to the species among Mycosis and Aerobic media. The Anaerobic medium was significantly inferior to Mycosis and Aerobic in the detection rate of *C. glabrata* and *C. parapsilosis*, but the detection of *S. cerevisiae* (45 %) did not differ significantly from Aerobic (65 %) and was much lower than Mycosis (90 %; *p* = 0.005).

**Table 2:** Positivity ratio of clinical blood samples cultured on Mycosis, Aerobic, and Anaerobic media

Species	No. of sets	Mycosis		Aerobic		Anaerobic		*p*-value, Fisher’s exact test		
		No. of vials Y/Y+B/B^a^	%^b^	No. of vials Y/Y+B/B^a^	%^b^	No. of vials Y/Y+B/B^a^	%^b^	M/AE	M/AN	AE/AN
*C. glabrata*	20	15/0/0	75	12/2/1	70	1/3/2	15	1.0000	**0.0020**	**0.0011**
*C. parapsilosis*	15	11/1/1	80	10/3/1	86	2/3/0	33.3	1.0000	**0.0253**	**0.0078**
*S. cerevisiae*	11	10/0/0	90.9	5/2/4	63.63	5/0/3	45.5	0.3108	**0.0052**	0.6699
*C. albicans*	3	2/0/0	66	2/0/0	66	0/0/0	0			
*C. tropicalis*	2	2/0/0	100	1/0/1	50	0/0/1	0			
Total	51	40/1/1	80.39	30/7/7	72.5	8/5/6	25.49	0.4843	**0.0000**	**0.0000**

^a^Number of vials positive for: *Y* yeasts, *Y+B* yeasts and bacteria, *B* bacteria

^b^Percentage of vials positive for fungi, including mixed fungal-bacterial cultures

*M/AE* comparison between Mycosis and Aerobic in terms of positivity to yeasts

*M/AN* comparison between Mycosis and Anaerobic in terms of positivity to yeasts

*AE/AN* comparison between Aerobic and Anaerobic in terms of positivity to yeasts

The medium TTD of pure fungal cultures was lower on Mycosis than on Aerobic media (50 h vs. 78 h, *p* = 0.001), especially for *C. glabrata* (28.76 h vs. 86 h) and *S. cerevisiae* (53 h vs. 102 h) (Table 3). It was observed that, in the 12 examination sets, fungi (*C. glabrata* 7 strains, *S. cerevisiae* 4, and *C. albicans* 1) were detected earlier on Mycosis than on Aerobic media (differences of 15 h to 5 days), while Aerobic detected *C. tropicalis* (1) and *C. parapsilosis* (6) faster than Mycosis media (difference of 1–24 h). Due to the low number of positive Anaerobic media, analysis of the TTD was performed only for *S. cerevisiae*, which was detected significantly faster on Anaerobic than on Aerobic media (*p* = 0.004).

**Table 3:** Time to detection (TTD; in hours) of fungi in clinical blood samples cultured on Mycosis (M), Aerobic (AE), and Anaerobic (AN) media (results for mixed fungal-bacterial cultures are not included)

Species	Mycosis		Aerobic		Anaerobic		*p*-value^a^		
	No. of positive vials	TTD (medium ± SD)	No. of positive vials	TTD (medium ± SD)	No. of positive vials	TTD (medium ± SD)	M/AE	M/AN	AE/AN
*C. glabrata*	15	29.22 ± 11.48	12	86 ± 40	1	35.9	**0.0000**		
*C. parapsilosis*	11	65 ± 47.33	10	83 ± 37	2	76 ± 28.2	0.6290		
*S. cerevisiae*	10	53.6 ± 9.18	5	107.26 ± 57.8	5	71.08 ± 9.1	**0.0108**	**0.004**	0.204
*C. albicans*	2	24.74	2	39.29	0	0			
*C. tropicalis*	2	20.63	1	15.63	0	0			
Total	40	50 ± 31.6	30	78.7 ± 41.1	8	67.9 ± 18.27	**0.0012**	0.1198	0.4769

^a^Student’s *t*-test for differences between means for independent variables

Mixed fungal-bacterial cultures were obtained in seven Aerobic (*C. glabrata* and *Enterococcus faecium* 2, *C. parapsilosis* and *E. coli* 2, *C. parapsilosis* and *A. baumannii* 1, *S. cerevisiae* and *K. pneumoniae* 1, *S. cerevisiae* and *S. epidermidis* 1), six Anaerobic (*C. glabrata* and *E. faecium* 2, *C. glabrata* and *E. coli* 1, *C. parapsilosis* and *E. coli* 3), and one Mycosis medium (*C. parapsilosis* and *E. coli*). The mixed samples signaled positivity within 24 h (mean 11.35 ± 8.65 h on Aerobic and 13.14 ± 3.9 h on Anaerobic). Among the vials negative for fungi, bacterial growth was found in seven Aerobic (*S. epidermidis* 2, *E. coli* 2, *S. aureus* 1, *S. haemolyticus* 1, and *Acinetobacter baumannii* 1), six Anaerobic (*S. epidermidis* 3, *E. coli* 2, *S. aureus* 1), and one Mycosis medium (*Acinetobacter baumannii*).

## Discussion

The data obtained in this study prove that both Aerobic and Mycosis media serve as the right conditions for the culture of pathogenic fungi and that the TTD in this media is related to the species. The analysis of the performance of blood culture systems and media in clinical settings represents the most relevant validation test. Nevertheless, such an analysis is often difficult, due to many factors, which could have an influence on the recovery of fungi from the blood, e.g., fluctuations in microbe density, presence of antibiotics and coexisting bacterial infection, as well as the technical accuracy of the test performance (blood volume, time of placement in the compartment, etc.). Most of these factors could easily be controlled via simulated, laboratory-prepared blood cultures, which have been used by many investigators [5–11]. In the presented study, both methods were applied. The culture of fungi in the same conditions (the same amount of blood and yeast inoculum) on three BACTEC media illustrated the capacity of the BACTEC system. The strains originated from clinical blood samples and were collected in the mycological laboratory of the Department of Microbiology of the Medical University of Wrocław within a period of 7 years. The diversity in the number of strains representing particular species was connected to the frequency of infection. Unfortunately, we did not routinely use Mycosis media and two other hospitals were invited to study the clinical usefulness of BACTEC media. The data provided were limited to examination sets performed simultaneously on these three media; nevertheless, they included almost all cases of occurring fungemia and reflect specific epidemiological problems that arise, namely the high proportion of infection due to *Saccharomyces cerevisiae* in Center I and infection due to *C. parapsilosis* in Center II.

It is well known that many fungal pathogens are characterized by slow multiplication and, for this reason, traditional mycological practice has mandated at least 10–14 days incubation time for blood cultures [12]. Therefore, it is important that, in our study, all isolates were tested in simulated blood cultures, but only one isolate of *C. neoformans* was detected on Mycosis as well as Aerobic media within 5 days—the time usually accepted for bacterial blood cultures. In clinical samples, the longest incubation required to obtain positivity was 7 days, and it pertains to only two cases. These data support recommendations of the EFISG (at least 5 days incubation) [3] and the algorithm described recently by Bosshard, who proposed 5–7 days of incubation of fungal blood cultures, except for dimorphic fungi [12].

There are some discrepancies between reports about the positivity ratio and false results obtained in simulated cultures with BACTEC media, especially BACTEC Anaerobic, which is a medium not designed for fungi. Some authors [8, 9], who reported a positivity of Aerobic as high as 90–98 %, and Anaerobic ranging between 10 and 15 %, found that almost all vials which were signaled by the BACTEC system as negative proved to be positive after terminal subculture. Conversely, according to the data collected by Klingspor et al. [6], there was no growth observed after the subculture of negative vials; thus, there was no false negativity. In the current study, all isolates used to prepare simulated cultures were grown on Aerobic and Mycosis, but the positivity ratio of Anaerobic media was low (9 %). We observed that some negative Anaerobic bottles (11 %) were positive in subcultures, of which a high proportion was *C. glabrata* (12 out of 19; 63 %). Importantly, the number of fungal colonies growing after the subculture of false-negative vials was low (1–10 CFU). It is clear that the condition served in Anaerobic media poorly support the growth of inoculated fungi, and that, in rare cases, despite the negative signal of the BACTEC system, a limited fungal cell load may be present. In the case of clinical blood samples, the false-negative results are regarded rather seldom, but were reported in some media of the BACTEC as well as BacT/ALERT systems [13, 14]. Interestingly, in the polymicrobial model of infection, detection of yeasts by Anaerobic media was enhanced up to 79 % [6]. This rule does not apply to Aerobic media. A similar experiment performed by Cateau et al. [11] with the media of the BacT/ALERT system (bioMérieux), led to opposite results and conclusions; that is, bacteria could hide the development of fungi. Also, Meyer et al. [15], who analyzed the results of clinical blood cultures, found that the detection of fungi from a mixed fungal-bacterial infection in an Aerobic medium was substantially reduced compared to cases from monoinfection (27 % vs. 75 %). The authors showed that, in such an infection, the use of Mycosis, a medium inhibiting the growth of most bacteria, significantly enhanced the probability of recovering fungi. The data cited above once more argue for the necessity to use different types of blood culture media in clinical practice. The advantage of the application of specialized mycological media is evident when analyzing the TTD of typical and rare etiological factors of fungemia. The most relevant difference in the TTD pertains to *C. glabrata*, which, in our experimental and clinical samples, similarly to other authors, was mostly detected within 24 h in Mycosis and after 2–3 days in Aerobic media [5–7]. It is also worthwhile to notice a complementary role of Anaerobic media, whose ability to detect *C. glabrata* is higher compared to other fungal species and the detection time is reliably shorter than in Aerobic media (22–40 h, depending on the study) [8, 9]. The exceptional results reported by Park et al. [10] showed that the Aerobic medium spiked with *C. glabrata* remained negative, whereas the Anaerobic medium was 100 % positive. Recently, Cobos-Trigueros et al. [16] analyzed the detection of *C. glabrata* on BACTEC Aerobic and Anaerobic media (without Mycosis) in a large number of candidemia cases. The authors found that the presence of *C. glabrata* can be predicted on the basis of exclusive or earlier detection of fungal growth in Anaerobic vials, but the method was characterized by low sensitivity, in the range 31–38 %. In our study, the usefulness of an Anaerobic medium in the manner described above (i.e., Anaerobic will be positive earlier than Aerobic) was supported by 1 out of 11 cases of fungemia due to *C. glabrata*. Instead, the Mycosis medium enables the early detection of *C. glabrata* in nine patients, in four of which it was the only positive medium. *C. glabrata* represents a frequent and emerging pathogen, less susceptible or resistant to azoles and associated with high (29 %) mortality [3, 4, 17]. The distribution of *C. glabrata* depends on the hospital department (hematology, ICU) and is also geographically related [3, 4, 17, 18]. In many European countries, including Poland, *C. glabrata* is the second cause of candidemia [18, 19]. The rapid detection and identification of this pathogen is extremely important for introducing proper therapy. It is clear that the use of Mycosis in patients with a suspected *C. glabrata* infection should be especially recommended. Recently, Tapia et al. [20] indicated that it is possible to identify ICU patients at risk of developing *C. glabrata* candidemia (less than 7 days in the hospital, abdominal surgery, fluconazole use, or absence of renal failure). Due to the medical and economical reasons that apply to Mycosis together with standard Aerobic and Anaerobic media, it may not always be accepted. The finding of Tapia et al. [20] illustrates that it is possible to narrow down the group of patients who are at the highest risk and should be tested with specialized mycological media.

The data obtained from simulated blood cultures presented in this study and those reported by Fricker-Hidalgo et al. [5] suggest that a very important indication for the application of the Mycosis medium should also be cases with suspected cryptococcosis. *Cryptococcus neoformans* is a species responsible for meningitis, which is characterized by delayed growth on typical media. The TTD of *C. neoformans* on Mycosis is also late (55 h), but significantly faster than in Aerobic media (78 h). Unfortunately, due to a low frequency of cryptococcosis, this species has, so far, been poorly represented in the clinical studies evaluating blood culture media. The comparison of Mycosis and Aerobic media performed previously by other authors [5] indicated statistically earlier detection in Mycosis than in Aerobic media not only for *C. glabrata* and *C. neoformans*, which was confirmed in this study, but also for *C. albicans*, *C. parapsilosis*, or *C. krusei*. In the presented study, the TTD of *C. albicans* and *C. parapsilosis* on Mycosis were also shorter than on Aerobic media, but the difference was not significant. In contrast, *C. krusei* were detected 1.5–2 h quicker on Aerobic than on Mycosis media, and this result was statistically significant. The clinical relevance of such a small difference is doubtful, especially since the TTD of *C. krusei* is relatively short in both media (19–21 h). The above discrepancies suggest the presence of significant intraspecies variability in the TTD existing in some fungal species. The usefulness of BACTEC media in a clinical sitting was analyzed in several studies [4, 15, 21, 22]. The published data are consistent in the sense that the positivity ratio and TTD are better on Mycosis than on Aerobic media. As an example, Chiarini et al. [21] found that Mycosis detected 77.4 %, Aerobic 71 %, and Anaerobic 8 % of fungal infections. What is puzzling, however, is that, in our data as well as in those described by Chiarini et al., a fourth of isolates were detected only on Mycosis media. It may be, in part, connected with the suppressive role of bacteria, which were present in more than 10 % of fungi-negative Aerobic and Anaerobic media. Another important factor affecting the results of blood cultures is the presence of antifungals. According to Köck et al. [7], Aerobic media showed better performance than Mycosis in blood samples comprising antimycotics, especially caspofungin or amphotericin B. Also, in the study by Ericson et al. [22], the TTD on Mycosis was significantly shorter than in BacT/Alert FA (bioMérieux) vials in patients who did not receive antifungal therapy, whereas in blood samples taken during therapy, the positivity of Mycosis vials was reduced. Taking into account the above listed limitations, it is understood that the usefulness of Mycosis may not always be evident when analyzing a single medical ward and a small patient population. The blood samples analyzed in this study were taken mostly before introducing the initial therapy, and are, thus, similar to those described by Ericson et al. [22]. We did not observe a difference in the positivity of Mycosis and Aerobic but the TTD on Mycosis was significantly better than on Aerobic media. It is worthwhile noting that *Saccharomyces cerevisiae* were detected even 2 days earlier on Mycosis than on Aerobic media.

In summary, we conclude that the addition of a Mycosis medium to the standard set of Aerobic and Anaerobic vials improves the sensitivity of the detection of fungi and reduces the detection time, which is dramatically high in infection due to *Candida glabrata*, *Cryptococcus neoformans*, and *Saccharomyces cereviasiae*. The Mycosis and Aerobic media are characterized by different properties, the most important among them being a better performance of Mycosis in mixed polymicrobial infection and a better performance of Aerobic in the presence of antifungals. Moreover, fungi showed notable inter- and intraspecies variability in the preference of particular media, which, in practice, complements each other. The simultaneous application of both Mycosis and Aerobic media offers the possibility to detect a broader number of isolates and improve the timing of the diagnosis.
